# 1536. Hepatitis C Care Cascade Before and After Lifted Insurance Restrictions In Birth Cohort and Non-Birth Cohort Patients

**DOI:** 10.1093/ofid/ofac492.091

**Published:** 2022-12-15

**Authors:** Nathan Aleger, Nathan Aleger, Nathan Aleger, Melissa O Jenkins, Meliisa Jenkins, Michael Gielach, Brittany Battle, Ann A Avery

**Affiliations:** MetroHealth, Cleveland, OH; MetroHealth, Cleveland, OH; MetroHealth, Cleveland, OH; MetroHealth, Cleveland, OH; MetroHealth, Cleveland, OH; MetroHealth, Cleveland, OH; Case Western Reserve University School of Medicine, Cleveland, Ohio

## Abstract

**Background:**

In 2016, the MetroHealth System implemented a system-wide hepatitis C (HCV) screening initiative in novel settings. We previously showed that those born 1945–1965 (birth cohort, BC) had higher rates of linkage to care (LTC) and sustained virologic response (SVR) rates compared to non-BC (not born 1945–1965). Since 2019, restrictions on fibrosis, sobriety and prescriber have been loosened. We hypothesized that gaps in care cascade between BC and non-BC patients (including LTC rates) would lessen in those diagnosed after 2019.

**Methods:**

We compared the HCV LTC rates and care cascade in patients diagnosed from Jan 1, 2016 to June 30 2017 (early group) to those diagnosed July 1, 2019 to Dec 31, 2020 (late group) as well between BC and non-BC patients. We performed logistic regression to evaluate the impact of race, gender, known prior HCV positivity, site of HCV diagnosis, HIV, insurance, active injection drug use (IDU), remote IDU, psychiatric disorder, BC status and early/late diagnosis on LTC.

**Results:**

The study population comprised 2103 HCV+ patients (1349 non-BC, 754 BC). There were 1026 patients in the early group and 1077 patients in the late group. 1353 patients were HCV RNA+. Of those, 914 (67.6%) were white, 306 (22.6%) African American; 872 (64.4%) male; 990 (74.5%) Medicaid; 822 (60.7%) outpatient diagnosis. On multivariable analysis, factors affecting.

LTC were: BC (aOR 1.71, 95%CI 1.19–2.45, *p*=0.003); diagnosed inpatient (aOR 0.45 (0.32–0.66), p< 0.0001), emergency department (aOR 0.55, (0.35–0.89), *p*=0.0142) or jail (aOR 0.23, (0.13–0.4), *p*=0.23); active IDU (aOR 0.22, (0.14–0.35),p< 0.0001); remote IDU (aOR 0.60, (0.4–0.89)*p*=0.01); and diagnosis in the late group (aOR 0.67, (0.51–0.8), p=0.005). The care cascade improved in the late group, but gaps remained in non-BC vs BC (Table).

**Conclusion:**

The non-BC continued to have lower rates of LTC compared to BC in the late group.Overall LTC was decreased in the late group for both non-BC and BC likely due to COVID-19. However, improvement in medication approval was shown in the late group, and most patients who were prescribed treatment completed it. Active IDU remained a barrier to non-LTC, but did not fully explain the differences between BC and non-BC patients.

**Disclosures:**

**Melissa O. Jenkins, Meliisa Jenkins**, Gilead Sciences: Grant/Research Support.

